# Severe Asthma and Allergy: A Pediatric Perspective

**DOI:** 10.3389/fped.2019.00028

**Published:** 2019-02-11

**Authors:** Stefania Arasi, Federica Porcaro, Renato Cutrera, Alessandro G. Fiocchi

**Affiliations:** ^1^Pediatric Allergology Unit, Bambino Gesù Hospital (IRCCS), Rome, Italy; ^2^Pediatric Pulmonology & Respiratory Intermediate Care Unit, Sleep and Long-Term Ventilation Unit, Department of Pediatrics, Bambino Gesù Children's Hospital, Rome, Italy

**Keywords:** allergy, allergic rhinitis, atopic dermatitis, children, difficult-to-treat asthma, food allergy, severe asthma

## Abstract

Severe asthma in children is associated with significant morbidity and lung function decline. It represents a highly heterogeneous disorder with multiple clinical phenotypes. As its management is demanding, the social and economic burden are impressive. Several co-morbidities may contribute to worsen asthma control and complicate diagnostic and therapeutic management of severe asthmatic patients. Allergen sensitization and/or allergy symptoms may predict asthma onset and severity. A better framing of “allergen sensitization” and understanding of mechanisms underlying progression of atopic march could improve the management and the long-term outcomes of pediatric severe asthma. This review focuses on the current knowledge about interactions between severe asthma and allergies.

## Introduction

Atopic sensitization is a well-established, but not exclusive, risk factor for severe asthma both in children (1, 2) and adults (3), all over the world (1–4). Although its role in determining asthma severity has been considered limited in the past years, some reports confirm that allergy may play a significant role especially in childhood, when early atopic sensitization is crucial to determine the severity of disease.

Though most asthmatic children achieve symptoms' control through occasional bronchodilator (BD) use or low to medium dose of inhaled corticosteroids (ICSs), a small but significant subset of patients remains with uncontrolled asthma despite treatment with high-dose inhaled glucocorticoids (Table 1) or requiring such a treatment to remain well-controlled (5). This group of children with chronic symptoms and episodic exacerbation requiring short-acting beta_2_ agonists (SABA) is defined as affected by “difficult-to-treat asthma.” This definition includes poorly-controlled asthma due to at least one of the following: an incorrect diagnosis; comorbidities; poor adherence to therapy because of adverse psychological or environmental factors (6). “Severe asthma” is considered a specific subset of “difficult-to-treat asthma.” It is characterized by the need of higher intensity therapy in order to maintain symptom control or uncontrolled symptoms despite such therapy (6), proper diagnosis (7), and management of comorbidities and correction of unsuitable behavior for control disease.

**Table 1:** High-dose ICD dosages for children (mcg/d) according to Global Initiative for Asthma (GINA) guidelines.

**Drug name**	**GINA (6–11 y)**	**GINA (>12 y)**
Beclomethasone dipropionate (HFA)	>200	>400
Budesonide (DPI)	>400	>800
Budesonide (nebules)	>1,000	
Ciclesonide (HFA)	>160	>320
Fluticasone propionate (DPI)	>400	>500
Fluticasone propionate (HFA)	>500	>500
Mometasone furoate (DPI)	>440	>440

*DPI, dry powder inhalers; HFA, hydrofluoroalkane*.

In 2014, a task force of the European Respiratory Society (ERS) and the American Thoracic Society (ATS) updated the definition of severe asthma in pediatric patients. According to the latter, children affected by severe asthma require treatment with high-dose ICSs and either a long-acting beta-agonist (LABA) or a leukotriene antagonist for the previous year or systemic corticosteroids for at least 50% of the previous year to prevent uncontrolled asthma or asthma that remains uncontrolled despite this therapy (5).

In a birth cohort study, the prevalence of severe asthma has been estimated about 0.5 and 4.5% in all 10-year-olds and current asthmatic children when assessed in 10 year olds, respectively (8). Notwithstanding, it is associated with a significant economic burden related to more and severe symptoms needing of adjunctive medical resource use and higher health costs (9). Furthermore, the increased number of parent's working days lost during child asthma exacerbations is accompanied by less global economic productivity (10).

Since only a small percentage of asthmatic patients is affected by severe asthma, this clinical entity is still poorly known even if associated with notable morbidity. Both in children and adults, severe asthma is a heterogeneous disorder with multiple clinical phenotypes (11). However, elegant cluster analyses have shown that the role of atopic sensitization might be more important in the pathogenesis of severe asthma specifically in childhood onset asthma: more than 85% of children with severe asthma are severely atopic (12). In contrast, severe adult-onset asthma is a distinct phenotype that is usually not characterized by atopic sensitization, but often associated with nasal polyposis and sputum eosinophilia (13, 14). A brief overview of characteristics and differences between pediatric and adult-onset severe asthma is provided in Table 2.

**Table 2:** Characteristics of severe bronchial asthma in children and adults.

**Characteristics**	**Pediatric asthma**	**Adult-onset asthma**
IgE-sensitization	+++	+
Poly-sensitization	+++	+
High specific IgE levels	+++	+
Clinical heterogeneity (i.e., multiple phenotypes)	+++	+++
Severe non-allergic obese female prevalent phenotype	–	+++
Severe non-allergic eosinophilic phenotype (nasal polyposis, sputum eosinophilia, and aspirin sensitivity)	–	+++

Though it is well-recognized that atopic sensitization is an important risk factor mainly for pediatric asthma, the role of allergy in children affected by severe asthma is still under debate. This review aims to focus the role of allergy in pediatric severe asthma.

## The Atopic March

Though atopic manifestations may persist for several years and then resolve over time (15), in atopic children, adolescents, and adults allergy manifestations may evolve according to a predetermined sequence, characterized by the progression from atopic dermatitis (AD) to allergic rhinitis (AR) and asthma (16–18).

Therefore, it seems that atopic predisposition represents a major risk factor for developing all atopic diseases in patients for which the progression from AD to asthma defines the well-known “atopic march” (19). However, the temporal presentation of allergic diseases may differ from the usual progression of the atopic march due to genetic influences and environmental factors (20).

Foremost, allergens may penetrate easier a defective skin barrier, therefore leading to transcutaneous sensitization and subsequentially initiating the atopic march (21). Indeed, IgE sensitization to food or airborne allergens is a significant cofactor to induce the progression of the atopic march in patients with AD (22–24). Moreover, it is widely described that the risk of developing asthma in patients with AD is strictly related to both the clinical expression of IgE sensitization and the severity of eczema (25–27).

**Figure 1:**
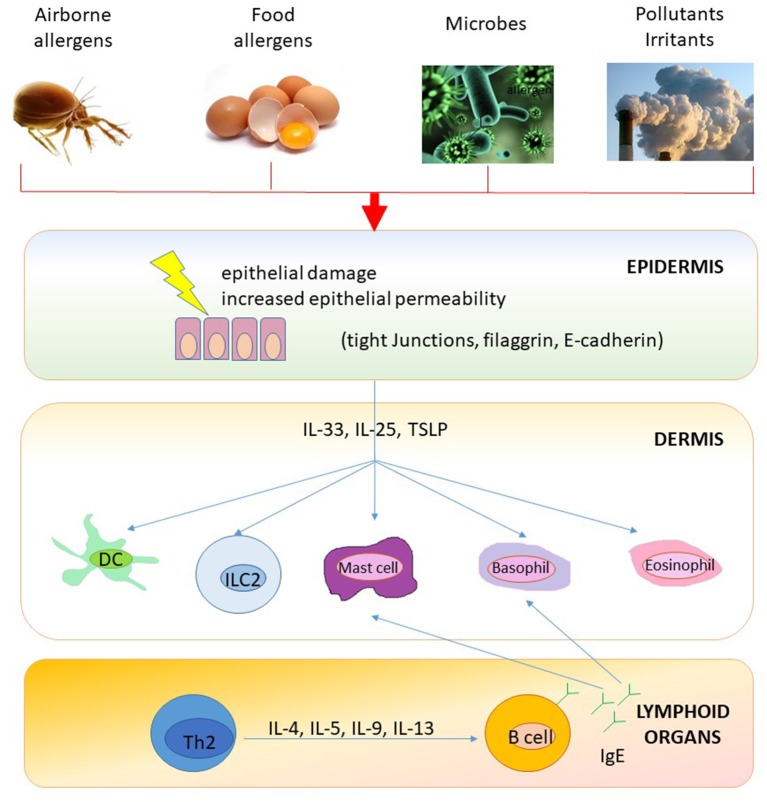
A model of epithelial barrier damage and skin IgE-sensitization. Impaired skin barrier (e.g., eczema) promotes foreign antigen (e.g., airborne and food allergens) penetration and activation of innate and specific immune responses. Epithelial cell-derived cytokines (such as TSLP, IL-33, and IL-25) license antigen presenting cells (i.e., dendritic cells) to drive type 2 immune responses and stimulate several cell types (including basophils, eosinophils, mast cells, and ILCs) to start and maintain allergic inflammation also in regional draining lymph nodes (e.g., B-cell IgE skewing). Furthermore, T cells circulate back to infiltrate the skin or are distributed peripherally to other end organs to initiate diverse atopic disorders. DC, dendritic cells; Ig, Immunoglobulin; IL, interleukin; ILC, Innate Lymphoid cells; Th, T helper cells; TSLP, thymic stromal lymphopoietin.

It seems that transcutaneous IgE-sensitization may precede airway sensitization (28) and that IgE-associated AD might represent the first step of the atopic march and, therefore, it may predict the upcoming development of allergic diseases, including food allergy, AR, and asthma (please see Figure 1) (28). Since AR is a further major risk factor for bronchial hyper-reactivity and asthma, it can precede asthma onset in the natural history of the atopic march (29–31).

## Allergic Comorbidities

**Figure 2:**
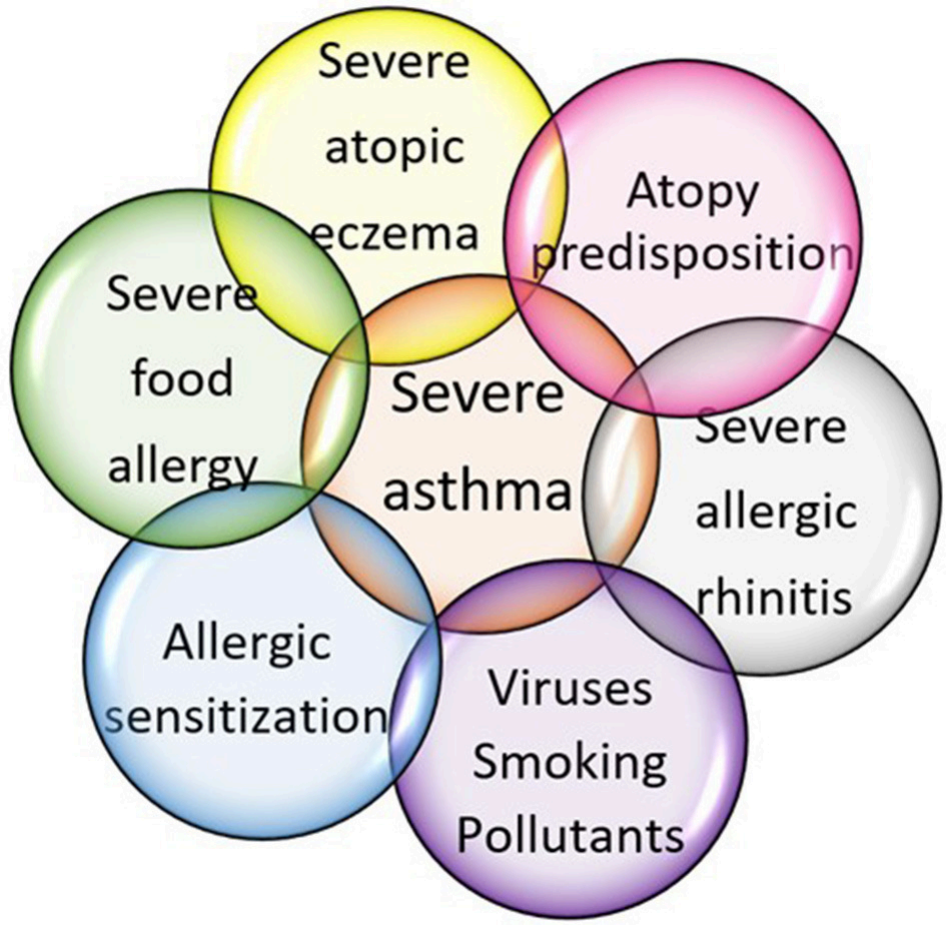
Factors contributing to severe asthma development in childhood.

Several factors and co-morbidities may contribute to worsen asthma control and complicate diagnostic and therapeutic management of severe asthmatic patients (Figure 2) (32).

### Atopic Dermatitis

Several studies reported that family history of atopy, early onset AD, higher initial severity of atopic eczema, hens' egg sensitization and male sex are associated with an increased risk of asthma in childhood (33). Furthermore, the percentage of patients with severe asthma and concomitant eczema is greater than expected and close relationship between asthma and atopic dermatitis severity has been reported (34).

Therefore, as AD and the subsequent atopic march mostly present in early infancy, primary, and secondary prevention should be attempted as early as possible to prevent asthma symptoms onset (35).

### Allergic Rhinitis

Allergic rhinitis is almost ubiquitous in children with asthma living in urban areas. The presence of allergic sensitization to inhalant allergens and rhinitis symptoms is typically associated with early onset of severe asthma (36). Patients with AR report poorer asthma control, more exacerbations and emergency visits (37) and have more difficulty in achieving symptom control (38).

Perennial allergic rhinitis with seasonal exacerbations is considered the most severe phenotype and most likely to be associated with difficult-to-control asthma (39).

This means that treating coexisting allergic rhinitis could improve asthma control and reduce healthcare resource utilization (40).

### Food Allergy

Respiratory symptoms as clinical manifestation of IgE-mediated food allergy (FA), usually, occur immediately after exposure to the offensive food and are accompanied by skin and/or gastrointestinal manifestations. Food allergen exposure occurs usually by ingestion, but the inhalation of food proteins (through dust or aerosolized particles) may also trigger respiratory symptoms (41).

Among patients with FA, asthmatic symptoms are more frequent in children, and especially in those with concomitant atopic dermatitis. In addition to respiratory symptoms occurring as a presentation of FA, patients with FA are at increased risk for developing asthma and often severe asthma as expression of progression of the atopic march. Symptomatic FA and food allergen sensitization are associated with asthma development both in younger and older children: it was reported that this association is stronger among children with multiple or severe FAs (42–44). Moreover, children with FA develop asthma earlier and at a higher prevalence than children without FA. The opposite is also true, as asthma is a risk factor for the persistence of food allergy (45–47).

Food allergy and food sensitization can be also considered as important markers to predict asthma severity. Indeed, it is reported that children with FA and sensitization to at least one food (e.g., egg, milk, soy, peanut, wheat, and fish) had worse lung function (48), higher rates of hospitalization, emergency department visits, use of systemic glucocorticoids (49), or need of mechanical ventilation for severe asthma exacerbation (50, 51).

### Aspirin Sensitization

Aspirin-exacerbated respiratory disease (AERD) is a chronic medical condition, usually in adults and adolescents, consisting of three clinical features: sinus disease with recurrent nasal polyps, asthma, and sensitivity to aspirin and other non-steroidal anti-inflammatory drugs (NSAIDs). Even though asthma is not always present in AERD, asthma symptoms develop 1–3 years after the development of rhinitis or later. When present, asthma is severe and difficult to treat and often characterized by increased residual volume and diminished diffusing capacity due to increased airway remodeling (52).

## Role of Atopic Sensitization in Severe Asthma

Previous studies showed that atopic sensitization is a major risk factor for severe asthma in children (53–55). Overall, the expression “atopic sensitization” refers to the positivity either of serum allergen-specific IgE (sIgE) or a positive skin prick test (SPT) to allergen extracts. Arbitrary cut-off points have been set: levels of sIgE >0.35 KU/l (56) and a mean wheal diameter ≥3 mm (SPT) (57). Though these tests are highly sensitive, their mere positivity do not mean itself clinical disease. Quantification of atopic sensitization increases the specificity in relation to childhood asthma presence and severity (4), and asthma persistence in adulthood (58–60). The severity of asthma correlates with both sIgE levels and the number of sensitizations, also on the molecular level. The number of component-specific sensitizations correlate with disease severity in **grass** allergic children (61) as well as in **house dust mites** allergic (HDM) pediatric patients. In the Multicentre Allergy Study (MAS) cohort, a birth cohort started in 1990 in Germany, the number of HDM-component specific sensitizations increased with disease severity and with age. Sensitization to Der p 1 and Der p 23 before the age of 5 years was predictive of asthma at school age (62). Similarly, in the Manchester Asthma and Allergy Study (MAAS) birth cohort, asthmatic children were characterized by more complex molecular patterns of IgE sensitization to grass and mite molecules (Der p 1 and Der p 2) (63). In a French study, atopic sensitization to Der p 2 and Der f 2 was more common in severe asthma. In a cohort of 300 asthmatic children (age range, 4–12 years), higher levels of Der p 1 and pet allergen [cat (Fel d 1), dog (Can f 1)] were found to be associated with greater asthma severity (64). Similarly, in **latex** allergy sensitization to 3 (5, 6.01/6.02) of the 12 recombinant natural rubber antigens so far known was strongly linked with asthma (65).

### Fungal Allergy

Fungal allergy drives asthma severity, too (66). Sensitization to molds has been estimated: 7–20% in the general asthma population; 35–75% in severe asthma patients; 54–91% in life-threatening asthma population (67–69). The severity of exacerbation relates to the different fungi species: in particular, Aspergillus or Alternaria or *Cladosporium* spp. sensitization has been linked to severe asthma (70, 71).

Long-term or uncontrolled fungal infections are associated with a poor controlled asthma, bronchiectasis, and chronic allergic bronchopulmonary aspergillosis (ABPA) (71). The term “Severe asthma with fungal sensitization” (SAFS), introduced by Denning et al. (71), describes a specific phenotype in patients with persistent severe asthma (despite standard treatment) and evidence of fungal sensitization, and do not meet the criteria for ABPA. An EAACI Task Force sets the total IgE cut-off at <1,000 IU/ml for SAFS and >1,000 IU/ml for ABPA, a specific endotypes of asthma, with a genetic predisposition.

## Role of Viruses, Smoking, and Pollutants

In poli-sensitized asthmatics, daily exposure to allergens combined with other enhancing factors, such as viral infections, smoking (even tertiary one), and/or environmental pollution, influences the asthma course and severity (Figure 2). There is robust evidence concerning the synergistic effect of **viral lower respiratory tract infections** (LRTI) and IgE sensitization on asthma development, particularly in children predisposed to atopy (72) and asthma exacerbation (73). Increased risks of asthma inception in atopic predisposed children include: the type of virus (more than 10-fold increased risk for asthma development with rhinovirus compared to 5-fold with respiratory syncytial virus); the severity of viral LRTI; and the age during viral LRTI (74). The risk of hospital admission due to asthma exacerbation is increased by the interaction among respiratory viral infections in combination with atopic sensitization and exposure to allergens (75).

### Cigarette Smoking

Cigarette smoking itself may influence asthma severity, through different patho-mechanisms (76). There is evidence that smoking increases itself serum IgE levels, especially in male adults (77), and rises the risk of IgE sensitization, mainly to occupational allergens (78). Nevertheless, in severe asthmatic patients, the complex association between cigarette smoking and allergy remains currently controversial.

Data reports that children exposed to **air pollution** are at major to develop IgE sensitization to inhalant allergens (79, 80). However, the immunological mechanisms underlying this link remain to be better clarified. Notwithstanding, some data suggest that ultrafine carbon black particles may induce maturation of dendritic cells *in vitro* (81), which might then facilitate sensitization to airborne allergens. On the other side, airborne pollutants may modulate the inflammatory cellular response in the lungs, thereby lowering the threshold for sensitization.

## Prevention Strategies

As widely described above and resumed in Table 2, children with early onset atopy, high specific IgE-sensitization and multiple IgE-sensitizations are at increased risk for developing severe asthma in childhood. In addition, it is well-established that severe allergic diseases more frequently coexist (Figure 2).

Based on these premises, it is reasonable that prevention strategies and proper treatments of atopic diseases could prevent occurrence of severe asthma and viceversa. However, asthma development depends on complex and still not fully known interception of genes and environment. Therefore, effective primary prevention strategies for asthma, and especially for severe asthma in children, -though highly desirable- might be difficult to be identified both at population and individual level.

### Primary Prevention

Some studies have suggested that maternal consumption of allergenic food (such us cow's milk, peanut, or fish) and vitamin D and E intake during pregnancy could be associated to decreased risk of allergy and wheezing in their offspring, respectively (82–84).

Conflicting results have been reported about the beneficial effect of maternal breastfeeding on pediatric asthma development (85). The role of prebiotics, probiotics, and synbiotic interventions (86) and other dietary supplements (such as nucleosides and nucleotides) (87) is under investigation. Overall, the level of evidence remains currently low or even very low because of the risk of bias, heterogeneity among studies, imprecision, and inconsistency of results, as well as indirectness of available research.

Instead, there is stronger evidence concerning maternal smoking and tobacco post-natal exposition. They are both associated to increased risk of asthma in offspring (88).

### Atopic Dermatitis

Since the epithelium plays an important role in protecting against the development of allergic diseases and the occurrence of transcutaneous IgE-sensitization may precede airway sensitization (Figure 1), proactive emollient therapy able to make stronger the epithelial barrier may prevent or delay the development of IgE-sensitization in children affected by AD (89).

### Allergic Rhinitis

It is well-known that AR and asthma often coexist. As they share genetic background, chronic airway inflammation pathway and similar triggers (allergen exposure, viral infections, cold air, and air pollution) (90), treatment of rhinitis can be beneficial for preventing severe asthma exacerbations.

The role of exposure to airborne allergens on AR and asthma is well-established and strict avoidance of the culprit allergen(s) is desirable though it is often hard or even impossible. Symptomatic drugs for AR, such as H1-antihistamines and intranasal corticosteroids are not recommended for asthma management (40). However, data suggest that the use of H1-antihistamines in AR children is associated with delayed asthma development (91) and improvement of asthma outcomes (92). Similarly, a significant reduction of asthma symptom scores and rescue medication use has been reported for patients with AR and coexisting asthma by using intranasal corticosteroid therapy (93). Moreover, anti-leukotrienes target both upper and lower airways and could be beneficial in patients with asthma and concomitant AR (94).

Biological drugs—such as anti-IgE therapy (i.e., omalizumab) and antibody against the α-subunit of receptor for IL-4 and IL-13 (i.e., dupilumab)- have been approved for a few specific phenotypes of severe asthmatic patients at different ages (Table 3). They could be beneficial on both diseases: severe asthma and severe AR (95, 96). Notwithstanding, allergen immunotherapy (AIT) is considered the only etiological treatment able to prevent asthma development (97), to improve asthma symptoms in AR affected children (98), and to prevent new sensitizations in already sensitized patients (99). Furthermore, since adverse events are more common during the escalation or build-up phases of AIT, omalizumab has been suggested as “add-on therapy” to AIT (100). Nevertheless, larger studies are needed to identify patients who would benefit the addition of omalizumab to AIT, as well as optimal dosing strategies and duration treatment (101).

**Table 3:** Main biological targeted treatments for severe asthma in children.

**Drug (trade name), dosage**	**Mechanism of action**	**Suggested population**	**Adverse effects**
**APPROVED**			
**Omalizumab** (Xolair), s.c. injections every 2–4 wks, depending on body weight and IgE levels	Anti- IgE; binds Fc receptor of free circulating IgE and downloads IgE production	Age >6 yrs; 30 UI < IgE < 700 UI^*^; positive skin test or elevated specific IgE level toward a perennial	Anaphylaxis (~0.2% pts); monitor for helmintic infection
**Mepolizumab** (Nucala), 100 mg in pts aged 12 yrs or older (40 mg in pts aged 6–11 yrs) by s.c. injections every 4 wks	Anti- IL-5; binds circulating IL-5	Age >12 yrs; eosinophlic asthma	Zoster (rare); avoid if active helminithic infection
**UNDER INVESTIGATION**			
**Reslizumab** (Cinqair), approved for adults (3 mg/kg by i.v. injections every 4 wks)	Anti- IL-5; binds circulating IL-5	Eosinophilic asthma	Anaphylaxis (rare); avoid if active helmintic infection
**Dupilumab** (Dupixent), approved for adults with atopic dermatitis	Anti- IL-4 and anti-IL-13; binds common α-subunit of receptor for IL-4 and IL-13	Eosinophlic asthma	Eosinophilia (rare); avoid live vaccines; avoid if active helmintic infection

IL, interleukin; i.v., intravenous; pt, patient; s.c., subcutaneous; wk, week; yr, year.

* *Upper limit varies according to body weight and regulatory authorities*.

### Food Allergy

Strict avoidance of the culprit food(s) represents currently the standard therapeutic option for FA (102). However, accidental exposure is possible and related to severe adverse events. Allergen specific immunotherapy (AIT) alone or combined with adjuvants (including probiotics and anti-IgE monoclonal antibodies) is nowadays the only active treatment for FA (103). Nevertheless, it is highly demanding, especially in patients with a severe phenotype, who usually have allergies to multiple foods and concomitant severe asthma. For these severe patients, biologicals might be a better potential therapy alone or in combination with AIT (104, 105). However, more data are needed.

### AERD

Careful avoidance of aspirin and other NSAIDs is mandatory in patients affected by AERD in order to prevent asthmatic exacerbations. The use of acetaminophen or selective COX-2 inhibitors should be encouraged as alternative drugs. Furthermore, the addition of a leukotriene receptor antagonist (e.g., montelukast) and 5-lipoxygenase inhibitors (e.g., zileuton) to standard asthma treatment have been shown to be effective in improving asthma outcomes (106). Aspirin desensitization is currently the only causative treatment in AERD affected patients improving both upper and lower airway symptoms; however, only a very small percentage of patients benefit from this therapeutic option (107). Biologic therapies (such as anti-IgE, anti-IL-5 monoclonal antibodies, IL-4α receptor antagonist, and anti-thymic stromal lymphopoietin) could be promising therapeutic options for AERD patients given their effectiveness in nasal polyposis and asthma (108).

Unfortunately, there is currently lack of data about any specific role of the treatment of allergic comorbidities in preventing the development of severe asthma in atopic children.

## Optimizing Treatment of Severe Asthma

The current guideline-based drug therapy of pediatric severe asthma is based primarily on data extrapolated from adult studies. High dose of inhalant **corticosteroids** (or oral corticosteroids) combined with a second controller (such as a LABA or leukotriene modifier/theophylline) are the mainstay of treatment (5).

However, the current challenge facing physicians and researchers is to provide a “**personalized medicine**,” which is tailored to the diverse patho-mechanisms underlying clinical presentations (109).

### Allergen Specific Immunotherapy

AIT has been demonstrated to have beneficial effects in the management of childhood allergic asthma, including effects on symptom control, medication use, and airway hyperresponsiveness (110). However, trials involve children with mild-moderate allergic asthma, and studies specifically examining the efficacy of AIT in children with severe asthma are missing. Notwithstanding, the majority of studies involved monosensitized patients, whereas most children with severe asthma are polysensitized, mainly in Southern Europe. In addition, allergen immunotherapy should be commenced in patients with well-controlled asthma, a situation which is less common among children with severe asthma. Since 2003, several **targeted therapies** have been approved for severe asthma and others are still under investigation (Table 3). Furthermore, they could be beneficial in other allergic comorbidities. However, their cost is often expensive. Therefore, a precise identification and selection of good responders is pivotal.

A **regular longitudinal assessment** of outcomes of children with severe asthma is pivotal. Follow-up appointments should be devoted not only to reduce maintenance therapy to the minimal amount required to achieve control of asthma symptoms, but also to assess the atopic status of the patient and any concomitant atopic co-morbidities and, therefore, address any modifiable factors (including allergen exposure, basics of inhaler technique, and adherence).

## Conclusions

Asthma is a common disease in childhood with a minority of affected children having severe asthma. Several data suggest that allergies may play a key role in children with severe asthma. Many children with severe asthma have coexisting allergic disease(s). Allergies to foods, molds, pollens, and pets have been associated with both asthma inception and severe asthma exacerbations. A better understanding of interactions between asthma and allergy and mechanistic implications of cofactors, such as virus infections, pollution, and smoking will allow the development of novel therapeutic targets and, therefore, additional strategies for improving disease control.

## Author Contributions

SA and FP wrote the first draft of the manuscript. AF and RC critically reviewed the manuscript. All authors read and approved the final manuscript.

### Conflict of Interest Statement

The authors declare that the research was conducted in the absence of any commercial or financial relationships that could be construed as a potential conflict of interest.

## Abbreviations

ABPA: allergic bronchopulmonary aspergillosis
AERD: aspirin exacerbated respiratory disease
AD: atopic dermatitis
AIT: allergen specific immunotherapy
AR: allergic rhinitis
ATS: American Thoracic Society
BD: bronchodilator
ERS: European Respiratory Society
FA: food allergy
HDM: house dust mites
ICS: inhaled corticosteroid
IgE: immunoglobulin E
LABA: long-acting beta2-agonist
MAAS: Manchester Asthma and Allergy Study
MAS: Multicentre Allergy Study
NSAIDs: non-steroidal anti-inflammatory drugs
SABA: short-acting beta2-agonist
SAFS: Severe asthma with fungal sensitization.

## References

[1] Addo-Yobo EO Custovic A Taggart SC Craven M Bonnie B Woodcock A. Risk factors for asthma in urban Ghana. J Allergy Clin Immunol. (2001)108:363–8. 10.1067/mai.2001.117464 11544454

[2] Al-Mousawi MS Lovel H Behbehani N Arifhodzic N Woodcock A Custovic A. Asthma and sensitization in a community with low indoor allergen levels and low petkeeping frequency. J Allergy Clin Immunol. (2004) 114:1389–94. 10.1016/j.jaci.2004.09.005 15577842

[3] Caminati M Pham DL Bagnasco D Canonica GW. Type 2 immunity in asthma. World Allergy Organ J. (2018) 11:13. 10.1186/s40413-018-0192-5 29988331 PMC6020328

[4] Stevens W Addo-Yobo E Roper J Woodcock A James H Platts-Mills T. Differences in both prevalence and titre of specific immunoglobulin E among children with asthma in affluent and poor communities within a large town in Ghana. Clin Exp Allergy (2011) 41:1587–94. 10.1111/j.1365-2222.2011.03832.x 21810123 PMC3505371

[5] Chung KF Wenzel SE Brozek JL Bush A Castro M Sterk PJ. International ERS/ATS guidelines on definition, evaluation and treatment of severe asthma. Eur Respir J. (2014) 43:343–73. 10.1183/09031936.00202013 24337046

[6] Guilbert TW Bacharier LB Fitzpatrick AM. Severe asthma in children. J Allergy Clin Immunol Pract. (2014) 2:489–500. 10.1016/j.jaip.2014.06.022 25213041 PMC4589165

[7] Gherasim A Dao A Bernstein JA. Confounders of severe asthma: diagnoses to consider when asthma symptoms persist despite optimal therapy. World Allergy Organ J. (2018) 11:29. 10.1186/s40413-018-0207-2 30459928 PMC6234696

[8] Lang A Carlsen KH Haaland G Devulapalli CS Munthe-Kaas M Mowinckel P. Severe asthma in childhood: assessed in 10-year olds in a birth cohort study. Allergy (2008) 63:1054–60. 10.1111/j.1398-9995.2008.01672.x 18691307

[9] Flórez-Tanus Á Parra D Zakzuk J Caraballo L Alvis-Guzmán N. Health care costs and resource utilization for different asthma severity stages in Colombia: a claims data analysis. World Allergy Organ J. (2018) 11:26. 10.1186/s40413-018-0205-4 30459927 PMC6231276

[10] Sullivan SD Rasouliyan L Russo PA Kamath T Chipps BE; TENOR Study Group Extent, patterns, and burden of uncontrolled disease in severe or difficult-to-treat asthma. Allergy (2007) 62:126–33. 10.1111/j.1398-9995.2006.01254.x 17298420

[11] Schatz M Hsu JW Zeiger RS Chen W Dorenbaum A Chipps BE Phenotypes determined by cluster analysis in severe or difficult-to-treat asthma. J Allergy Clin Immunol. (2014) 133:1549–56. 10.1016/j.jaci.2013.10.006 24315502

[12] Bossley CJ Fleming L Gupta A Regamey N Frith J Oates T. Pediatric severe asthma is characterized by eosinophilia and remodeling without T(H)2 cytokines. J Allergy Clin Immunol. (2012) 129:974–82. 10.1016/j.jaci.2012.01.059 22385633 PMC3381727

[13] Amelink M de Groot JC de Nijs SB Lutter R Zwinderman AH Sterk PJ. Severe adult-onset asthma: a distinct phenotype. J Allergy Clin Immunol. (2013) 132:336–41. 10.1016/j.jaci.2013.04.052 23806634

[14] Hekking PP Loza MJ Pavlidis S de Meulder B Lefaudeux D Baribaud F U-BIOPRED Study Group. Pathway discovery using transcriptomic profiles in adult-onset severe asthma. J Allergy Clin Immunol. (2018) 141:1280–90. 10.1016/j.jaci.2017.06.037 28756296

[15] Spergel JM. Epidemiology of atopic dermatitis and atopic march in children. Immunol Allergy Clin N Am. (2010) 30:269–80. 10.1016/j.iac.2010.06.003 20670812

[16] Bergmann RL Bergmann KE Lau-Schadensdorf S Luck W Dannemann A Bauer CP. Atopic diseases in infancy: the German Multicenter Atopy Study (MAS-90). Pediatr Allergy Immunol. (1994) 5:19–25. 10.1111/j.1399-3038.1994.tb00343.x 7728224

[17] Wahn U. What drives the allergic march? Allergy (2000) 55:591–99. 10.1034/j.1398-9995.2000.00111.x 10921457

[18] Høst A. Frequency of cow's milk allergy in childhood. Ann Allergy Asthma Immunol. (2002) 89:33–7. 10.1016/S1081-1206(10)62120-5 12487202

[19] Spergel JM Paller AS. Atopic dermatitis and the atopic march. J Allergy Clin Immunol. (2003) 112:S118–27. 10.1016/j.jaci.2003.09.033 14657842

[20] Ricci G Patrizi A Baldi E Menna G Tabanelli M Masi M. Long-term follow-up of atopic dermatitis: retrospective analysis of related risk factors and association with concomitant allergic diseases. J Am Acad Dermatol. (2006) 55:765–71. 10.1016/j.jaad.2006.04.064 17052480

[21] Hogan MB Peele K Wilson NW. Skin barrier function and its importance at the start of the atopic march. J Allergy (Cairo) (2012) 2012:901940. 10.1155/2012/901940 22619686 PMC3352623

[22] Warner JO A double-blinded, randomized, placebo-controlled trial of cetirizine in preventing the onset of asthma in children with atopic dermatitis: 18 months' treatment and 18 months' post-treatment follow-up. J Allergy Clin Immunol. (2001) 108:929–37. 10.1067/mai.2001.120015 11742270

[23] Novembre E Cianferoni A Lombardi E Bernardini R Pucci N Vierucci A. Natural history of “intrinsic” atopic dermatitis. Allergy (2001) 56:452–3. 10.1034/j.1398-9995.2001.056005452.x 11350313

[24] Wuthrich B Schmid-Grendelmeier P. Natural course of AEDS. Allergy (2002) 57:267–8. 10.1034/j.1398-9995.2002.1n3572.x 11906351

[25] Filipiak-Pittroff B Schnopp C Berdel D Naumann A Sedlmeier S Onken A. Predictive value of food sensitization and filaggrin mutations in children with eczema. J Allergy Clin Immunol. (2011) 128:1235–41. 10.1016/j.jaci.2011.09.014 22030464

[26] Marenholz I Kerscher T Bauerfeind A Esparza-Gordillo J Nickel R Keil T. An interaction between filaggrin mutations and early food sensitization improves the prediction of childhood asthma. J Allergy Clin Immunol. (2009) 123:911–6. 10.1016/j.jaci.2009.01.051 19348926

[27] Gustafsson D Sjöberg O Foucard T. Development of allergies and asthma in infants and young children with atopic dermatitis - a prospective follow-up to 7 years of age. Allergy (2000) 55:240–5. 10.1034/j.1398-9995.2000.00391.x 10753014

[28] Dohi M Okudaira H Sugiyama H Tsurumachi K Suko M Nakagawa T. Bronchial responsiveness to mite allergen in atopic dermatitis without asthma. Int Arch Allergy Immunol. (1990) 92: 138–42. 10.1159/000235204 2242928

[29] Leynaert B Neukirch C Kony S Guénégou A Bousquet J Aubier M. Association between asthma and rhinitis according to atopic sensitization in a population-based study. J Allergy Clin Immunol. (2004) 113:86–93. 10.1016/j.jaci.2003.10.010 14713912

[30] Ciprandi G Cirillo I Vizzaccaro A Milanese M Tosca MA. Airway function and nasal inflammation in seasonal allergic rhinitis and asthma. Clin Exp Allergy (2004) 34:891–6. 10.1111/j.1365-2222.2004.01970.x 15196276

[31] Spergel JM. Atopic march: link to upper airways. Curr Opin Allergy Clin Immunol. (2005) 5:17–21. 10.1097/00130832-200502000-00005 15643339

[32] Bonner K Roberts G. Does allergy explain why some children have severe asthma? Clin Exp Allergy (2018) 48:1594–605. 10.1111/cea.13234 30019503

[33] Amat F Soria A Tallon P Bourgoin-Heck M Lambert N Deschildre A. New insights into the phenotypes of atopic dermatitis linked with allergies and asthma in children: an overview. Clin Exp Allergy (2018) 48:919–34. 10.1111/cea.13156 29676818

[34] Lee JK Han D. Atopic dermatitis is an important comorbidity in severe asthma. Ann Allergy Asthma Immunol. (2018) 120:661–71. 10.1016/j.anai.2018.02.026 29496463

[35] Czarnowicki T Krueger JG Guttman-Yassky E. Novel concepts of prevention and treatment of atopic dermatitis through barrier and immune manipulations with implications for the atopic march. J Allergy Clin Immunol. (2017) 139:1723–34. 10.1016/j.jaci.2017.04.004 28583445

[36] Moore WC Meyers DA Wenzel SE Teague WG Li H Li X. Identification of asthma phenotypes using cluster analysis in the Severe Asthma Research Program. Am J Respir Crit Care Med. (2010) 181:315–23. 10.1164/rccm.200906-0896OC 19892860 PMC2822971

[37] Bousquet J Gaugris S Kocevar VS Zhang Q Yin DD Polos PG Increased risk of asthma attacks and emergency visits among asthma patients with allergic rhinitis: a subgroup analysis of the improving asthma control trial. Clin Exp Allergy (2005) 35:723–7. 10.1111/j.1365-2222.2005.02251.x 15969661

[38] Valovirta E Pawankar R. Survey on the impact of comorbid allergic rhinitis in patients with asthma. BMC Pulm Med. (2006) 6:S3. 10.1186/1471-2466-6-S1-S3 17140421 PMC1698496

[39] Togias A Gergen PJ Hu JW Babineau DC Wood RA Cohen RT. Rhinitis in children and adolescents with asthma: ubiquitous, difficult to control, and associated with asthma outcomes. J Allergy Clin Immunol. (2018) 10.1016/j.jaci.2018.07.041. [Epub ahead of print]. 30213627 PMC6408960

[40] Bousquet J Khaltaev N Cruz AA Denburg J Fokkens WJ Togias A. Allergic Rhinitis and its Impact on Asthma (ARIA) 2008. Allergy (2008) 63:8–160. 10.1111/j.1398-9995.2007.01620.x 18331513

[41] Sampson HA Aceves S Bock SA James J Jones S Lang D. Food allergy: a practice parameter update-2014. J Allergy Clin Immunol. (2014) 134:1016–25. 10.1016/j.jaci.2014.05.013 25174862

[42] Rhodes HL Sporik R Thomas P Holgate ST Cogswell JJ. Early life risk factors for adult asthma: a birth cohort study of subjects at risk. J Allergy Clin Immunol. (2001) 108:720–5. 10.1067/mai.2001.119151 11692095

[43] Pham MN Wang J. Management of food allergies and asthma in schools. Ann Allergy Asthma Immunol. (2018) 121:391–9. 10.1016/j.anai.2018.07.028 30290894

[44] Sherenian MG Singh AM Arguelles L Balmert L Caruso D Wang X. Association of food allergy and decreased lung function in children and young adults with asthma. Ann Allergy Asthma Immunol. (2018) 121:588–93. 10.1016/j.anai.2018.07.037 30081088 PMC6215513

[45] Fiocchi A. Incremental prognostic factors associated with cow's milk allergy outcomes in infant and child referrals: the Milan Cow's Milk Allergy Cohort study. Ann Allergy Asthma Immunol. (2008) 101:166–73. 10.1016/S1081-1206(10)60205-0 18727472

[46] Wood RA Sicherer SH Vickery BP Jones SM Liu AH Fleischer DM. The natural history of milk allergy in an observational cohort. J Allergy Clin Immunol. (2013) 131:805–12. 10.1016/j.jaci.2012.10.060 23273958 PMC3691063

[47] Sicherer SH Wood RA Vickery BP Jones SM Liu AH Fleischer DM. The natural history of egg allergy in an observational cohort. J Allergy Clin Immunol. (2014) 133:492–9. 10.1016/j.jaci.2013.12.1041 24636473 PMC3959659

[48] Friedlander JL Sheehan WJ Baxi SN Kopel LS Gaffin JM Ozonoff A. Food allergy and increased asthma morbidity in a School-based Inner-City Asthma Study. J Allergy Clin Immunol Pract. (2013) 1:479–84. 10.1016/j.jaip.2013.06.007 24058900 PMC3777668

[49] Wang J Visness CM Sampson HA. Food allergen sensitization in inner-city children with asthma. J Allergy Clin Immunol. (2005) 115:1076–80. 10.1016/j.jaci.2005.02.014 15867869

[50] Roberts G Patel N Levi-Schaffer F Habibi P Lack G. Food allergy as a risk factor for life-threatening asthma in childhood: a case-controlled study. J Allergy Clin Immunol. (2003) 112:168–74. 10.1067/mai.2003.1569 12847494

[51] Berns SH Halm EA Sampson HA Sicherer SH Busse PJ Wisnivesky JP. Food allergy as a risk factor for asthma morbidity in adults. J Asthma (2007) 44:377–81. 10.1080/02770900701364031 17613633

[52] Kennedy JL Stoner AN Borish L. Aspirin-exacerbated respiratory disease: prevalence, diagnosis, treatment, and considerations for the future. Am J Rhinol Allergy (2016) 30:407–13. 10.2500/ajra.2016.30.4370 28124651 PMC5108840

[53] Fitzpatrick AM Teague WG Meyers DA Peters SP Li X Li H. Heterogeneity of severe asthma in childhood: confirmation by cluster analysis of children in the National Institutes of Health/National Heart, Lung, and Blood Institute Severe Asthma Research Program. J Allergy Clin Immunol. (2011) 127:382–9. 10.1016/j.jaci.2010.11.015 21195471 PMC3060668

[54] Lee E Kim YH Cho HJ Yoon J Jung S Yang SI. Clinical phenotypes of bronchial hyperresponsiveness in school-aged children. Ann Allergy Asthma Immunol. (2018) 121:434–43. 10.1016/j.anai.2018.05.033 29886267

[55] Lu KD Phipatanakul W Perzanowski MS Balcer-Whaley S Matsui EC Atopy, but not obesity is associated with asthma severity among children with persistent asthma. J Asthma (2016) 53:1033–44. 10.3109/02770903.2016.1174259 27144330 PMC5066807

[56] Heinzerling LM Burbach GJ Edenharter G Bachert C Bindslev-Jensen C Bonini S. GA(2)LEN skin test study I: GA(2) LEN harmonization of skin prick testing: novel sensitization patterns for inhalant allergens in Europe. Allergy (2009) 64:1498–506. 10.1111/j.1398-9995.2009.02093.x 19772515

[57] Johansson SG Hourihane JO Bousquet J Bruijnzeel-Koomen C Dreborg S Haahtela T. A revised nomenclature for allergy. An EAACI position statement from the EAACI nomenclature task force. Allergy (2001) 56:813–24. 10.1034/j.1398-9995.2001.t01-1-00001.x 11551246

[58] Lodrup Carlsen KC Soderstrom L Mowinckel P Haland G Pettersen M Munthe Kaas MC Asthma prediction in school children; the value of combined IgE antibodies and obstructive airways disease severity score. Allergy (2010) 65:1134–40. 10.1111/j.1398-9995.2010.02344.x 20219060

[59] Sly PD Boner AL Bjorksten B Bush A Custovic A Eigenmann PA. Early identification of atopy in the prediction of persistent asthma in children. Lancet (2008) 372:1100–110. 10.1016/S0140-6736(08)61451-8 18805338 PMC4440493

[60] Soto-Quiros M Avila L Platts-Mills TA Hunt JF Erdman DD Carper H. High titers of IgE antibody to dust mite allergen and risk for wheezing among asthmatic children infected. J Allergy Clin Immunol. (2012) 129:1499–505. 10.1016/j.jaci.2012.03.040 22560151 PMC3792652

[61] Hatzler L Panetta V Lau S Wagner P Bergmann RL Illi S. Molecular spreading and predictive value of preclinical IgE response to Phleum pratense in children with hay fever. J Allergy Clin Immunol. (2012) 130:894–901. 10.1016/j.jaci.2012.05.053 22841010

[62] Posa D Perna S Resch Y Lupinek C Panetta V Hofmaier S. Evolution and predictive value of IgE responses toward a comprehensive panel of house dust mite allergens during the first 2 decades of life. J Allergy Clin Immunol. (2017) 139:541–9. 10.1016/j.jaci.2016.08.014 27793411

[63] Custovic A Sonntag HJ Buchan IE Belgrave D Simpson A Prosperi MCF. Evolution pathways of IgE responses to grass and mite allergens throughout childhood. J Allergy Clin Immunol. (2015) 136:1645–52. 10.1016/j.jaci.2015.03.041 25962900

[64] Gent JF Belanger K Triche EW Bracken MB Beckett WS Leaderer BP. Association of pediatric asthma severity with exposure to common household dust allergens. Environ Res. (2009) 109:768–74. 10.1016/j.envres.2009.04.010 19473655 PMC2706291

[65] Vandenplas O Froidure A Meurer U Rihs HP Rifflart C Soetaert S. The role of allergen components for the diagnosis of latex-induced occupational asthma. Allergy (2016) 71:840–9. 10.1111/all.12872 26940537

[66] Masaki K Fukunaga K Matsusaka M Kabata H Tanosaki T Mochimaru T. Characteristics of severe asthma with fungal sensitization. Ann Allergy Asthma Immunol. (2017) 119:253–7. 10.1016/j.anai.2017.07.008 28801088

[67] Larenas-Linnemann D Baxi S Phipatanakul W Environmental Allergens Workgroup. Clinical evaluation and management of patients with suspected fungus sensitivity. J Allergy Clin Immunol Pract. (2016) 4:405–14. 10.1016/j.jaip.2015.10.015 26755100

[68] Black PN Udy AA Brodie SM. Sensitivity to fungal allergens is a risk factor for life-threatening asthma. Allergy (2000) 55:501–4. 10.1034/j.1398-9995.2000.00293.x 10843433

[69] Vicencio AG Santiago MT Tsirilakis K Stone A Worgall S Foley EA. Fungal sensitization in childhood persistent asthma is associated with disease severity. Pediatr Pulmonol. (2014) 49:8–14. 10.1002/ppul.22779 23401301

[70] Tham R Dharmage SC Taylor PE Katelaris CH Vicendese D Abramson MJ. Outdoor fungi and child asthma health service attendances. Pediatr Allergy Immunol. (2014) 25:439–49. 10.1111/pai.12257 24902620

[71] Denning DW Pashley C Hartl D Wardlaw A Godet C Del Giacco S. Fungal allergy in asthma-state of the art and research needs. Clin Transl Allergy (2014) 4:14. 10.1186/2045-7022-4-14 24735832 PMC4005466

[72] Jackson DJ Gangnon RE Evans MD Roberg KA Anderson EL Pappas TE. Wheezing rhinovirus illnesses in early life predict asthma development in high-risk children. Am J Respir Crit Care Med. (2008) 178:667–72. 10.1164/rccm.200802-309OC 18565953 PMC2556448

[73] Kusel MM de Klerk NH Kebadze T Vohma V Holt PG Johnston SL. Early-life respiratory viral infections, atopic sensitization, and risk of subsequent development of persistent asthma. J Allergy Clin Immunol. (2007) 119:1105–10. 10.1016/j.jaci.2006.12.669 17353039 PMC7125611

[74] Martorano LM Grayson MH. Respiratory viral infections and atopic development: from possible mechanisms to advances in treatment. Eur J Immunol. (2018) 48:407–14. 10.1002/eji.201747052 29244204 PMC5844827

[75] Kim CK Callaway Z Gern JE. Viral infections and associated factors that promote acute exacerbations of asthma. Allergy Asthma Immunol Res. (2018) 10:12–17. 10.4168/aair.2018.10.1.12 29178673 PMC5705478

[76] Polosa R Thomson NC. Smoking and asthma: dangerous liaisons. Eur Respir J. (2013) 41:716–26. 10.1183/09031936.00073312 22903959

[77] Oryszczyn MP Annesi-Maesano I Charpin D Paty E Maccario J Kauffmann F. Relationships of active and passive smoking to total IgE in adults of the Epidemiological Study of the Genetics and Environment of Asthma, Bronchial Hyperresponsiveness, and Atopy (EGEA). Am J Respir Crit Care Med. (2000) 161:1241–6. 10.1164/ajrccm.161.4.9905027 10764318

[78] Cockcroft DW. Environmental causes of asthma. Semin Respir Crit Care Med. (2018) 39:12–18. 10.1055/s-0037-1606219 29427981

[79] Bowatte G Lodge CJ Knibbs LD Lowe AJ Erbas B Dennekamp M. Traffic-related air pollution exposure is associated with allergic sensitization, asthma, and poor lung function in middle age. J Allergy Clin Immunol. (2017) 139:122–9. 10.1016/j.jaci.2016.05.008 27372567

[80] Bowatte G Lodge C Lowe AJ Erbas B Perret J Abramson MJ. The influence of childhood traffic-related air pollution exposure on asthma, allergy and sensitization: a systematic review and a meta-analysis of birth cohort studies. Allergy (2015) 70:245–56. 10.1111/all.12561 25495759

[81] Brandt EB Biagini Myers JM Acciani TH Ryan PH Sivaprasad U Ruff B. Exposure to allergen and diesel exhaustparticles potentiates secondary allergen-specific memory responses, promoting asthma susceptibility. J Allergy Clin Immunol. (2015) 136:295–303. 10.1016/j.jaci.2014.11.043 25748065 PMC4530081

[82] Bunyavanich S Rifas-Shiman SL Platts-Mills TA Workman L Sordillo JE Camargo CA Jr. Peanut, milk, and wheat intake during pregnancy is associated with reduced allergy and asthma in children. J Allergy Clin Immunol. (2014) 133:1373–82. 10.1016/j.jaci.2013.11.040 24522094 PMC4004710

[83] Best KP Gold M Kennedy D Martin J Makrides M. Omega-3 long-chain PUFA intake during pregnancy and allergic disease outcomes in the offspring: a systematic review and meta-analysis of observational studies and randomized controlled trials. Am J Clin Nutr. (2016) 103:128–43. 10.3945/ajcn.115.111104 26675770

[84] Chawes BL Bonnelykke K Stokholm J Vissing NH Bjarnadóttir E Schoos AM. Effect of vitamin D3 supplementation during pregnancy on risk of persistent wheeze in the offspring: a randomized clinical trial. JAMA (2016) 315:353–61. 10.1001/jama.2015.18318 26813208

[85] Chan-Yeung M Becker A. Primary prevention of childhood asthma and allergic disorders. Curr Opin Allergy Clin Immunol. (2006) 6:146–51. 10.1097/01.all.0000225150.91661.34 16670504

[86] Forsberg A West CE Prescott SL Jenmalm MC. Pre- and probiotics for allergy prevention: time to revisit recommendations? Clin Exp Allergy (2016) 46:1506–21. 10.1111/cea.12838 27770467

[87] Hawkes JS Gibson RA Roberton D Makrides M. Effect of dietary nucleotide supplementation on growth and immune function in term infants: a randomized controlled trial. Eur J Clin Nutr. (2006) 60:254–64. 10.1038/sj.ejcn.1602310 16234834

[88] Burke H Leonardi-Bee J Hashim A Pine-Abata H Chen Y Cook DG. Prenatal and passive smoke exposure and incidence of asthma and wheeze: systematic review and meta-analysis. Pediatrics (2012) 129:735–44. 10.1542/peds.2011-2196 22430451

[89] Shaker M Murray RGP Mann JA. The ins and outs of an 'outside-in' view of allergies: atopic dermatitis and allergy prevention. Curr Opin Pediatr. (2018) 30:576–81. 10.1097/MOP.0000000000000646 29750772

[90] Bousquet J Van Cauwenberge P Khaltaev N Aria Workshop Group World Health Organization. Allergic rhinitis and its impact on asthma. J Allergy Clin Immunol. (2001) 108(Suppl. 5):S147–334. 10.1067/mai.2001.118891 11707753

[91] Bustos GJ Bustos D Bustos GJ Romero O. Prevention of asthma with ketotifen in preasthmatic children: a three-year follow-up study. Clin Exp Allergy (1995) 25:568–73. 10.1111/j.1365-2222.1995.tb01096.x 7648464

[92] Crystal-Peters J Neslusan C Crown WH Torres A. Treating allergic rhinitis in patients with comorbid asthma: the risk of asthma-related hospitalizations and emergency department visits. J Allergy Clin Immunol. (2002) 109:57–62. 10.1067/mai.2002.120554 11799366

[93] Lohia S Schlosser RJ Soler ZM. Impact of intranasal corticosteroids on asthma outcomes in allergic rhinitis: a meta-analysis. Allergy (2013) 68:569–79. 10.1111/all.12124 23590215

[94] Price DB Swern A Tozzi CA Philip G Polos P. Effect of montelukast on lung function in asthma patients with allergic rhinitis: analysis from the COMPACT trial. Allergy (2006) 61:737–42. 10.1111/j.1398-9995.2006.01007.x 16677244

[95] Humbert M Boulet LP Niven RM Panahloo Z Blogg M Ayre G. Omalizumab therapy: patients who achieve greatest benefit for their asthma experience greatest benefit for rhinitis. Allergy (2009) 64:81–4. 10.1111/j.1398-9995.2008.01846.x 19076535

[96] GINA 217 Available online at: https://ginasthma.org/wp-content/uploads/2018/04/wms-GINA-2018-report-V1.3-002.pdf

[97] Halken S Larenas-Linnemann D Roberts G Calderón M Angier E Pfaar O. EAACI Guidelines on Allergen Immunotherapy: Prevention of allergy. Pediatr Allergy Immunol. (2017) 28:728–45. 10.1111/pai.12807 28902467

[98] Kristiansen M Dhami S Netuveli G Halken S Muraro A Roberts G. Allergen immunotherapy for the prevention of allergy: a systematic review and meta-analysis. Pediatr Allergy Immunol. (2017) 28:18–29. 10.1111/pai.12661 27653623

[99] Nolte H Hébert J Berman G Gawchik S White M Kaur A. Randomized controlled trial of ragweed allergy immunotherapy tablet efficacy and safety in North American adults. Ann Allergy Asthma Immunol. (2013) 110:450–6. 10.1016/j.anai.2013.03.013 23706715

[100] Lombardi C Canonica GW Passalacqua G. Allergen immunotherapy as add-on to biologic agents. Curr Opin Allergy Clin Immunol. (2018) 18:502–8. 10.1097/ACI.0000000000000479 30148718

[101] Dantzer JA Wood RA. The use of omalizumab in allergen immunotherapy. Clin Exp Allergy (2018) 48:232–40. 10.1111/cea.13084 29315922

[102] Emons JAM Gerth van Wijk R. Food allergy and asthma: is there a link? Curr Treat Options Allergy (2018) 5:436–44. 10.1007/s40521-018-0185-1 30524933 PMC6244552

[103] Nurmatov U Dhami S Arasi S Pajno GB Fernandez-Rivas M Muraro A. Allergen immunotherapy for IgE-mediated food allergy: a systematic review and meta-analysis. Allergy (2017) 72:1133–47. 10.1111/all.13124 28058751

[104] Arasi S Mennini M Valluzzi R Riccardi C Fiocchi A. Precision medicine in food allergy. Curr Opin Allergy Clin Immunol. (2018) 18:438–43. 10.1097/ACI.0000000000000465 30015641

[105] Wood RA Kim JS Lindblad R Nadeau K Henning AK Dawson P. A randomized, double-blind, placebo-controlled study of omalizumab combined with oral immunotherapy for the treatment of cow's milk allergy. J Allergy Clin Immunol. (2016) 137:1103–10. 10.1016/j.jaci.2015.10.005 26581915 PMC5395304

[106] Lee RU Stevenson DD. Aspirin-exacerbated respiratory disease: evaluation and management. Allergy Asthma Immunol Res. (2011) 3:3–10. 10.4168/aair.2011.3.1.3 21217919 PMC3005316

[107] Cho K-S Soudry E Psaltis AJ Nadeau KC McGhee SA Nayak JV. Long-term sinonasal outcomes of aspirin desensitization in aspirin exacerbated respiratory disease. Otolaryngol Head Neck Surg. (2014) 151:575–81. 10.1177/0194599814545750 25118195

[108] Tsetsos N Goudakos JK Daskalakis D Konstantinidis I Markou K. Monoclonal antibodies for the treatment of chronic rhinosinusitis with nasal polyposis: a systematic review. Rhinology (2018) 56:11–21. 10.4193/Rhin17.156 29396960

[109] Arasi S Corsello G Villani A Pajno GB. The future outlook on allergen immunotherapy in children: 2018 and beyond. Ital J Ped. (2018) 44:80. 10.1186/s13052-018-0519-4 29996875 PMC6042356

[110] Dhami S Kakourou A Asamoah F Agache I Lau S Jutel M. Allergen immunotherapy for allergic asthma: a systematic review and meta-analysis. Allergy (2017) 72:1825–48. 10.1111/all.13208 28543086

